# A homologue of the fungal tetraspanin Pls1 is required for *Epichloë festucae* expressorium formation and establishment of a mutualistic interaction with *Lolium perenne*

**DOI:** 10.1111/mpp.12805

**Published:** 2019-04-22

**Authors:** Kimberly A. Green, Carla J. Eaton, Matthew S. Savoian, Barry Scott

**Affiliations:** ^1^ Institute of Fundamental Sciences Massey University Palmerston North New Zealand

**Keywords:** tetraspanin, *Epichloë festucae*, expressorium, symbiosis

## Abstract

*Epichloë festucae* is an endophytic fungus that forms a mutualistic symbiotic association with the grass host *Lolium perenne*. Endophytic hyphae exit the host by an appressorium‐like structure known as an expressorium. In plant‐pathogenic fungi, the tetraspanin Pls1 and the NADPH oxidase component Nox2 are required for appressorium development. Previously we showed that the homologue of Nox2, NoxB, is required for *E. festucae* expressorium development and establishment of a mutualistic symbiotic interaction with the grass host. Here we used a reverse genetics approach to functionally characterize the role of the *E. festucae* homologue of Pls1, PlsA. The morphology and growth of Δ*plsA* in axenic culture was comparable to wild‐type. The tiller length of plants infected with Δ*plsA* was significantly reduced. Hyphae of Δ*plsA* had a proliferative pattern of growth within the leaves of *L. perenne* with increased colonization of the intercellular spaces and the vascular bundles. The Δ*plsA* mutant was also defective in expressorium development although the phenotype was not as severe as for Δ*noxB*, highlighting potentially distinct roles for PlsA and NoxB in signalling through the NoxB complex. Hyphae of Δ*plsA* proliferate below the cuticle surface but still occasionally form an expressorium‐like structure that enables the mutant hyphae to exit the leaf to grow on the surface. These expressoria still form a septin ring‐like structure at the point of cuticle exit as found in the wild‐type strain. These results establish that *E. festucae* PlsA has an important, but distinct, role to NoxB in expressorium development and plant symbiosis.

## Introduction

In mammalian cells, membrane‐bound NADPH oxidase (Nox) flavoenzymes catalyse the reduction of dioxygen to superoxide anions using electrons provided by NADPH, which are then converted into further reactive oxygen species (ROS) (Lambeth, 2004; Sumimoto, 2008). Mammalian Nox complexes are generally composed of the integral membrane protein flavocytochrome b558, a heterodimer composed of the catalytic subunit gp91^phox ^and the adaptor protein p22^phox^; and the cytosolic regulatory components Rac1/2, p40^phox^, p47^phox^ and p67^phox^ which upon activation get recruited to the membrane to interact with gp91^phox ^and p22^phox^, enabling ROS production (Rastogi *et al.*, 2017). Fungi generally contain two homologues of gp91^phox ^(Nox1/NoxA and Nox2/NoxB), a Rac1/2 homologue (RacA), a p67^phox^ homologue (NoxR), and two proteins which share similar functions to p40^phox^ and p47^phox^ (Cdc24 and BemA) (Aguirre *et al.*, 2005; Lalucque and Silar, 2003; Takemoto *et al.*, 2006, 2007, 2011). In addition, the fungal Nox complexes have functional homologues of the adaptor protein p22^phox^, corresponding to NoxD and Pls1 for the NoxA and NoxB complexes, respectively (Lacaze *et al.*, 2015; Siegmund *et al.*, 2015; Zhao *et al.*, 2016). In many plant‐pathogenic fungi, ROS production by the NoxA and NoxB complexes is required for successful host penetration and host colonization (Scott, 2015). To gain entry into a host plant, the fungal pathogen *Magnaporthe oryzae* forms a dome‐shaped cell known as an appressorium that breaches the leaf cuticle by physical force through the formation of a narrow penetration peg at the base of the appressorium called the ‘appressorium pore’ (Wilson and Talbot, 2009). Similar appressorium‐like structures have been observed in *Botrytis cinerea* (Siegmund *et al.*, 2013) and *Colletotrichum lindemuthianum* (Veneault‐Fourrey *et al.*, 2005). In comparison, *Verticillium dahliae* hyphae differentiate into hyphopodia and penetrate host roots via penetration pegs (Zhao *et al.*, 2016). Although the saprobic fungus *Podospora anserina* is not a plant pathogen, visualization of cellulose utilization has shown that *P. anserina* hyphae make specialized structures similar to appressoria that are able to penetrate and breach cellophane (Brun *et al.*, 2009). In *Magnaporthe*, *NOX1* and *NOX2* are required for appressorium‐mediated cuticle penetration (Egan *et al.*, 2007). In *P. anserina*, *Nox1* and *NoxD* mutants share similar defects in hyphal anastomosis, fruiting body formation and appressorium‐like development on cellophane (Lacaze *et al.*, 2015); results that are consistent with Nox1 (NoxA) and NoxD being components of the same Nox (Nox1) complex as supported by the demonstration that NoxA and NoxD from *B. cinerea* physically interact *in vitro* (Siegmund *et al.*, 2015). In *M. oryzae* (Clergeot *et al.*, 2001; Egan *et al.*, 2007), *B. cinerea* (Gourgues *et al.*, 2004) and *C. lindemuthianum* (Veneault‐Fourrey *et al.*, 2005), Pls1 and Nox2 are required for formation of appressoria penetration pegs, suggesting that Pls1 is the adaptor for the Nox2 complex. This hypothesis is further supported by the demonstration that in the saprobic fungus *P. anserina* (Brun *et al.*, 2009) and the plant‐pathogenic fungus *V. dahliae* (Zhao *et al.*, 2016), Nox2 and Pls1 are required for appressoria‐like cellophane penetration and the *V. dahliae* Pls1 and NoxB physically interact *in vitro* (Zhao *et al.*, 2016). In *M. oryzae*, the F‐actin cytoskeleton, septins Sep3, Sep4 and Sep5, and the exocyst components Sec3, Sec5, Sec6, Sec8, Sec15, Exo70 and Exo84 are recruited to the base of the appressorium in a ring‐like structure (Dagdas *et al.*, 2012; Gupta *et al.*, 2015; Ryder *et al.*, 2013). In *M. oryzae*, Nox2 is required for septin‐mediated reorientation of the F‐actin ring within the appressorium in the initial stages of host penetration, while Nox1 is required for maintaining the cortical F‐actin network around the penetration pore for subsequent host colonization (Ryder *et al.*, 2013). A similar F‐actin, septin and exocyst component ring has been observed in *V. dahliae* hyphopodia penetration pegs (Zhao *et al.*, 2016; Zhou *et al.*, 2017).

In comparison to plant‐pathogenic fungi, the fungal endophyte *E. festucae* forms mutualistic associations with temperate *Festuca* and *Lolium* grass hosts (Leuchtmann *et al.*, 1994; Schardl, 1996). In vegetative plant tissues, hyphae grow within the intercellular space of host cells (Christensen *et al.*, 1997) and systemically colonize the leaf sheath, leaf blade and inflorescences (May *et al.*, 2008; Scott *et al.*, 2012). Hyphae grow by tip growth within the true stem of the grass, where they enter into the developing leaf blade and sheath tissues, and become attached to the host cell wall by an adhesive matrix (Christensen and Voisey, 2007; Christensen *et al.*, 2002). Intercalary growth allows attached hyphae to avoid mechanical shearing as the host cells expand around them (Christensen *et al.*, 2008). Hyphal growth is tightly coordinated with host development. Once host cells stop elongating, hyphae cease growing, but remain metabolically active and maintain a mutualistic association within the host (Christensen and Voisey, 2007; Tan *et al.*, 2001). *E. festucae* hyphae also grow epiphytically on the host plant (Christensen *et al.*, 1997; Leuchtmann *et al.*, 1994). Endophytic hyphae exit the host cuticle layer by an appressorium‐like structure known as an expressorium (Becker *et al.*, 2016) to form a restricted hyphal network on the leaf surface. These epiphytic hyphae may increase host resistance to fungal pathogens through ‘niche exclusion’ (Moy *et al.*, 2000). To form expressoria *E. festucae* endophytic hyphae first colonize the tight spaces between the epidermal plant cells where they make contact with the host cuticle, thereby triggering formation of a swollen hyphal compartment, which differentiates and penetrates the undersurface of the leaf (Becker *et al.*, 2016). The epiphytic hyphae which emerge on the leaf surface remain connected to the endophytic hyphal network but have a different cell wall structure; in endophytic hyphae chitin appears to be restricted to septa whereas epiphytic hyphae contain abundant chitin in the cell wall (Becker *et al.*, 2016). This difference in cell wall structure may allow endophytic hyphae of *E. festucae* to avoid eliciting a host defence response when growing inside the plant (Becker *et al.*, 2016). Similar to plant‐pathogenic fungi that form appressoria, the NoxA and NoxB complexes are required for differentiation of expressoria (Becker *et al.*, 2016) and maintenance of a mutualistic symbiotic interaction between *E. festucae* and its host (Takemoto *et al.*, 2006, 2011; Tanaka *et al.*, 2006, 2008). In comparison to mutualistic wild‐type interactions, the Δ*noxA*, Δ*noxB* and Δ*noxR* mutants all show antagonistic interactions with the host plant. Mutant hyphae proliferate within the intercellular spaces, colonize the vascular bundles of the leaves and grow as sub‐cuticular hyphae beneath the host cuticle.

Given the novelty of the expressorium structure in *E. festucae*‐host associations, the aim of this study was to determine whether the *E. festucae* homologue of Pls1, like NoxB (Becker *et al.*, 2016), is required for expressorium formation and establishment of a mutualistic symbiotic association. Using a reverse genetics approach, we investigated this hypothesis and further determined whether the septin Sep3 is arranged in a ring‐like structure in the expressorium, as observed in other fungi (Dagdas *et al.*, 2012; Ryder *et al.*, 2013; Zhao *et al.*, 2016).

## Results

### *E. festucae* contains a Pls1 homologue

To identify the *E. festucae* Pls1 homologue, a tBLASTn search of the wild‐type Fl1 (E894) genome sequence (Schardl *et al.*, 2013a) was carried out using *M. oryzae* Pls1 (MGG_12594) as the query sequence. This search identified the gene model EfM3.019170, subsequently named *plsA*, as the *E. festucae* homologue (Fig. S1A). *E. festucae* PlsA is predicted to be a 224 amino acid protein that shares 49% amino acid identity to *M. oryzae*, 60% identity to *F. graminearum*, 51% identity to *P. anserina*, and 50% identity to *N. crassa* and *S. macrospora* Pls1 homologues when aligned using ClustalW (Fig. S1C). Bioinformatic analysis using InterPro and TMHMM predicts that PlsA contains four trans‐membrane domains and a tetraspanin EC2 domain (Fig. S1B,C). In *S. macrospora*, the transcription factor PRO1 binds to the consensus sequence GGCGCTTA within the promoter regions of *nox1*, *pro41 (noxD)*, *pls1* and *nox2*, suggesting PRO1 regulates Nox complex gene expression during fruiting body formation (Steffens *et al.*, 2016). In *E. festucae,* the homologue of PRO1, ProA, binds to a motif identical to the *S. macrospora* PRO1 consensus sequence in the intergenic region of the divergently transcribed *esdC* and *EF320,* and in the promoter regions of *symB* and *symC* (Green *et al.*, 2017; Tanaka *et al.*, 2013). By aligning several Clavicipitaceae *plsA* promoter sequences, a putative ProA binding motif, which does not include the additional 3′ A of this consensus sequence, was identified 192 bp upstream of the *E. festucae plsA* ATG start site (Fig. S2). To determine whether ProA regulates *plsA* gene expression *in planta*, we examined the *E. festucae* RNAseq data sets generated from plants infected with wild‐type and Δ*proA* mutants (Eaton *et al.*, 2015). This analysis revealed that although the *plsA* promoter contains a putative ProA binding site, deletion of *proA* does not significantly alter *plsA* expression *in planta* (fold difference −1.08, corrected *P* < 0.05).

### Deletion of *plsA* does not affect *E. festucae* culture morphology

**Figure 1:**
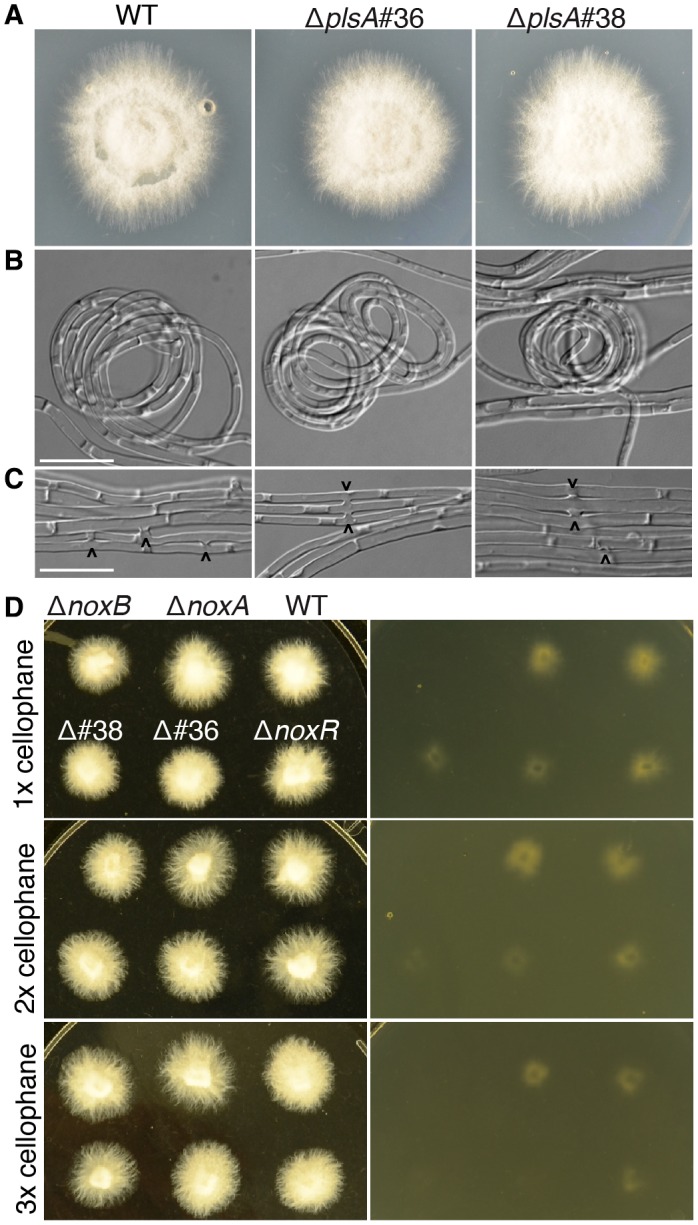
Culture morphology and growth phenotypes of wild‐type, Δ*plsA* and Nox complex mutant strains. (A) Colony morphology of wild‐type (WT) and ∆*plsA* (#36 and #38) cultures grown on PD agar for seven days at 22°C. (B) DIC images of cultures undergoing coil formation on 1.5% water agar after nine days of growth at 22°C. Scale bar = 20 μm. (C) DIC images of hyphae undergoing cell–cell fusion on 1.5% water agar after seven days of growth at 22°C. Fusion events are marked with arrowheads. Scale bar = 20 μm. (D) Cellophane penetration assay. Wild‐type and mutant strains were grown on PD medium overlaid with 1–3 cellophane layers (left). After seven days of growth at 22°C the cellophane layer was removed and mycelial growth on the underlying media photographed (right).

To investigate the role of PlsA in regulating hyphal morphology and growth, expressorium formation, and the establishment of a mutualistic symbiotic interaction between *E. festucae* and the host *Lolium perenne*, the *plsA* gene was deleted in the wild‐type strain background using a gene replacement approach (Fig. S3A). Protoplasts of wild‐type were transformed with a 3781 bp PCR amplified fragment containing the *plsA* deletion cassette and transformants selected using hygromycin. PCR screening and Southern blot analysis identified Δ*plsA*#36 and Δ*plsA*#38 as single copy ‘clean’ deletion mutants (Fig. S3B). These strains were subsequently selected for all further experiments. In axenic culture, hyphae of *E. festucae* wild‐type strain have a very distinct pattern of growth. Hyphal strands adhere to one another to form cables that extend outwards from the colony centre. Tip‐to‐side hyphal fusions occur frequently within these cables, and asexual conidiation and hyphal coiling are sparse (Becker *et al.*, 2016; Kayano *et al.*, 2013; Tanaka *et al.*, 2013). Several *E. festucae* mutants, including Δ*noxA*, Δ*noxR*, Δ*symB*, Δ*symC*, Δ*mobC*, Δ*proA*, Δ*mpkA* and Δ*mkkA*, exhibit a loss or reduction of hyphal fusion and increased conidiation in culture (Becker *et al.*, 2015, 2016; Green *et al.*, 2016, 2017; Kayano *et al.*, 2013; Tanaka *et al.*, 2013). To determine whether deletion of *plsA* results in a similar culture phenotype to these mutants, cultures of wild‐type and Δ*plsA* were grown on potato dextrose and water agar. No differences were observed in the overall radial growth, hyphal morphology, cell–cell fusion or coil formation between wild‐type and Δ*plsA* cultures (Fig. 1A–C), as was previously reported for the Δ*noxB* mutant (Kayano *et al.*, 2013).

### Deletion of *plsA* affects *E. festucae* growth on cellophane membranes

Several fungi have been documented as forming appressoria‐like penetration pegs or needle‐like structures in culture that allow hyphae to penetrate or degrade cellophane membranes (Brun *et al.*, 2009; Gu *et al.*, 2015; Gua *et al.*, 2014; Kucheryav *et al.*, 2008; Rispail and Pietro, 2009; Zhao *et al.*, 2016). Furthermore, several *nox2* and *pls1* mutants have been shown to exhibit cellophane penetration or degradation defects (Brun *et al.*, 2009; Gu *et al.*, 2015; Gua *et al.*, 2014; Kucheryav *et al.*, 2008; Rispail and Pietro, 2009; Zhao *et al.*, 2016). To determine whether the *E. festucae* NoxA and NoxB complexes or the tetraspanin PlsA have similar or distinct roles in allowing *E. festucae* hyphae to penetrate or degrade cellophane, we examined wild‐type, Δ*noxA*, Δ*noxB*, Δ*noxR* and Δ*plsA* hyphal growth on cellophane membranes. In comparison to wild‐type, Δ*noxA* and Δ*noxR* hyphae, which breached one to three layers of cellophane after five days of growth, both the Δ*plsA* and Δ*noxB* hyphae were impaired in their ability to breach cellophane, with the latter completely defective (Fig. 1D).

### PlsA is required for expressorium development and mutualistic symbiotic interaction

**Figure 2:**
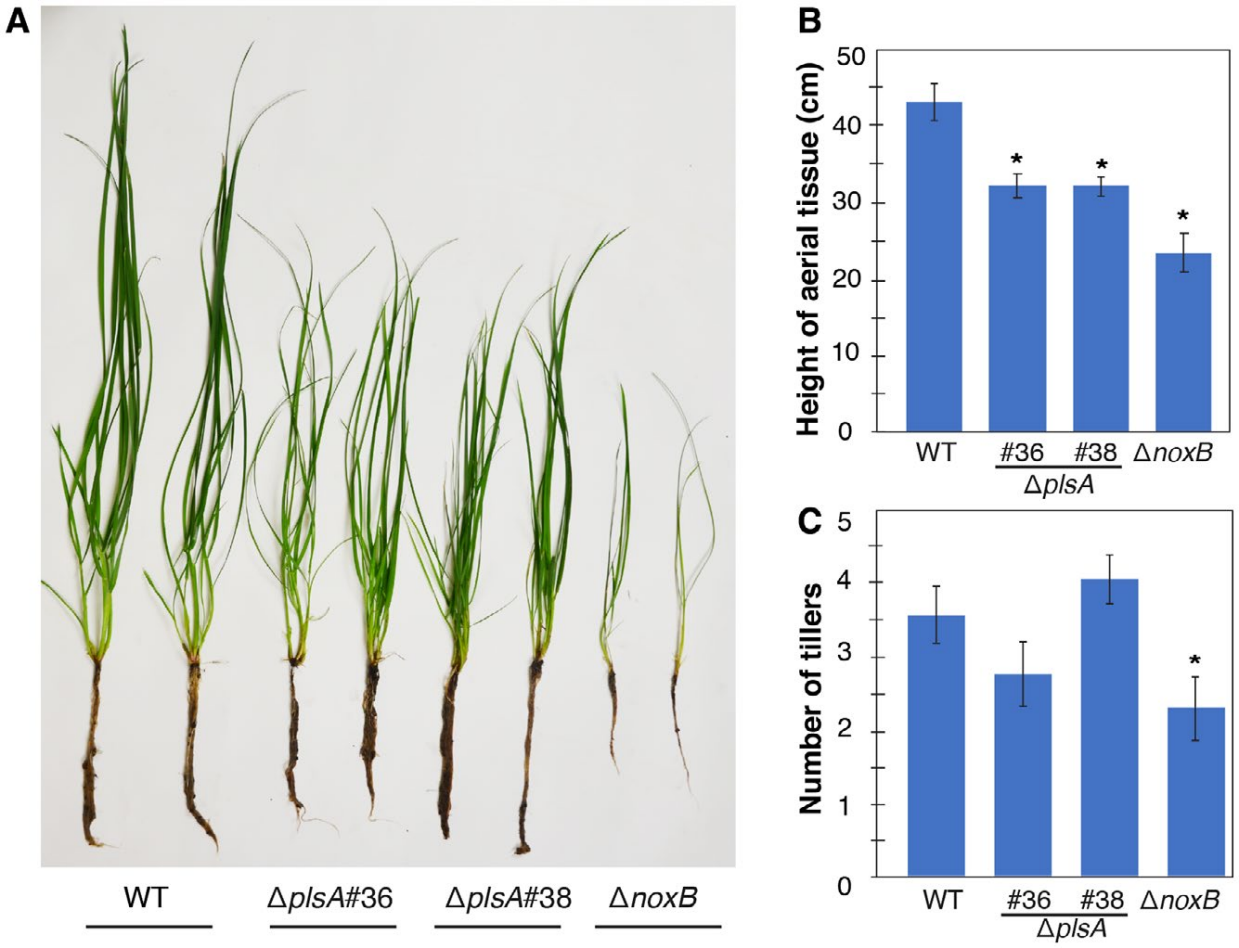
*In planta* host phenotypes of wild‐type and mutant strains. (A) Host phenotype of plants infected with *E. festucae* wild‐type (WT), ∆*plsA* and ∆*noxB* strains at eight weeks post planting. (B, C) Height of the tallest infected tiller and number of tillers (wild‐type and ∆*plsA* associations *n* = 14–18; ∆*noxB* associations *n* = 3). An asterisk indicates significant differences from wild‐type (*P* < 0.05), as determined by one‐way ANOVA test. Results are representative of three independent experiments.

**Figure 3:**
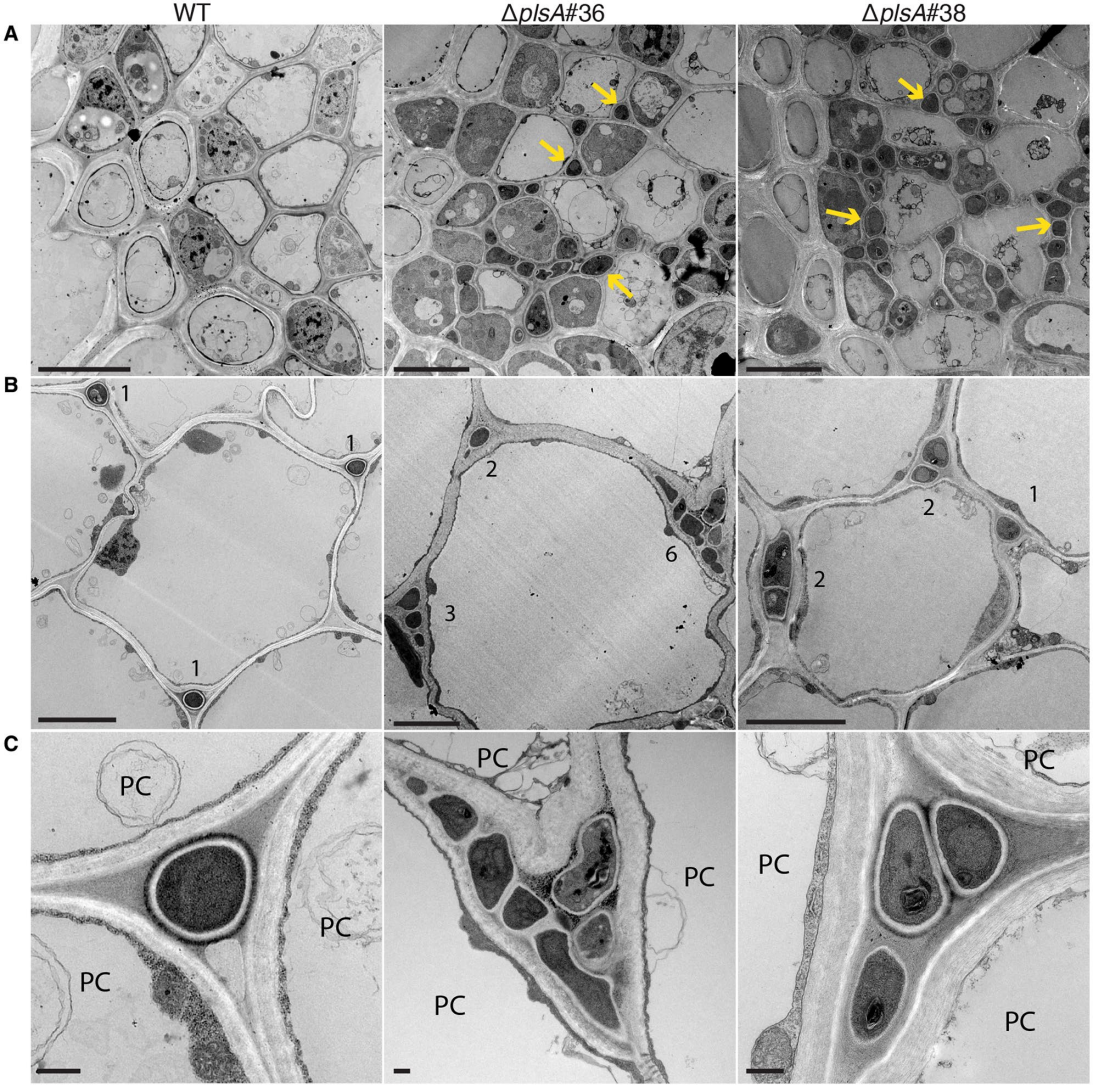
Transmission electron micrographs of *L. perenne* pseudostem cross‐sections infected with wild‐type (WT) and ∆*plsA* strains. (A) Vascular bundle colonization by ∆*plsA* but not WT. Example hyphae are indicated by yellow arrows. Bar = 5 μm. (B) Hyphae growing between plant cells. Numbers indicate the number of hyphae observed. Bar = 5 μm. (C) Higher magnification images of hyphae within the intercellular spaces of plant cells (PC). Bar = 500 nm.

**Figure 4:**
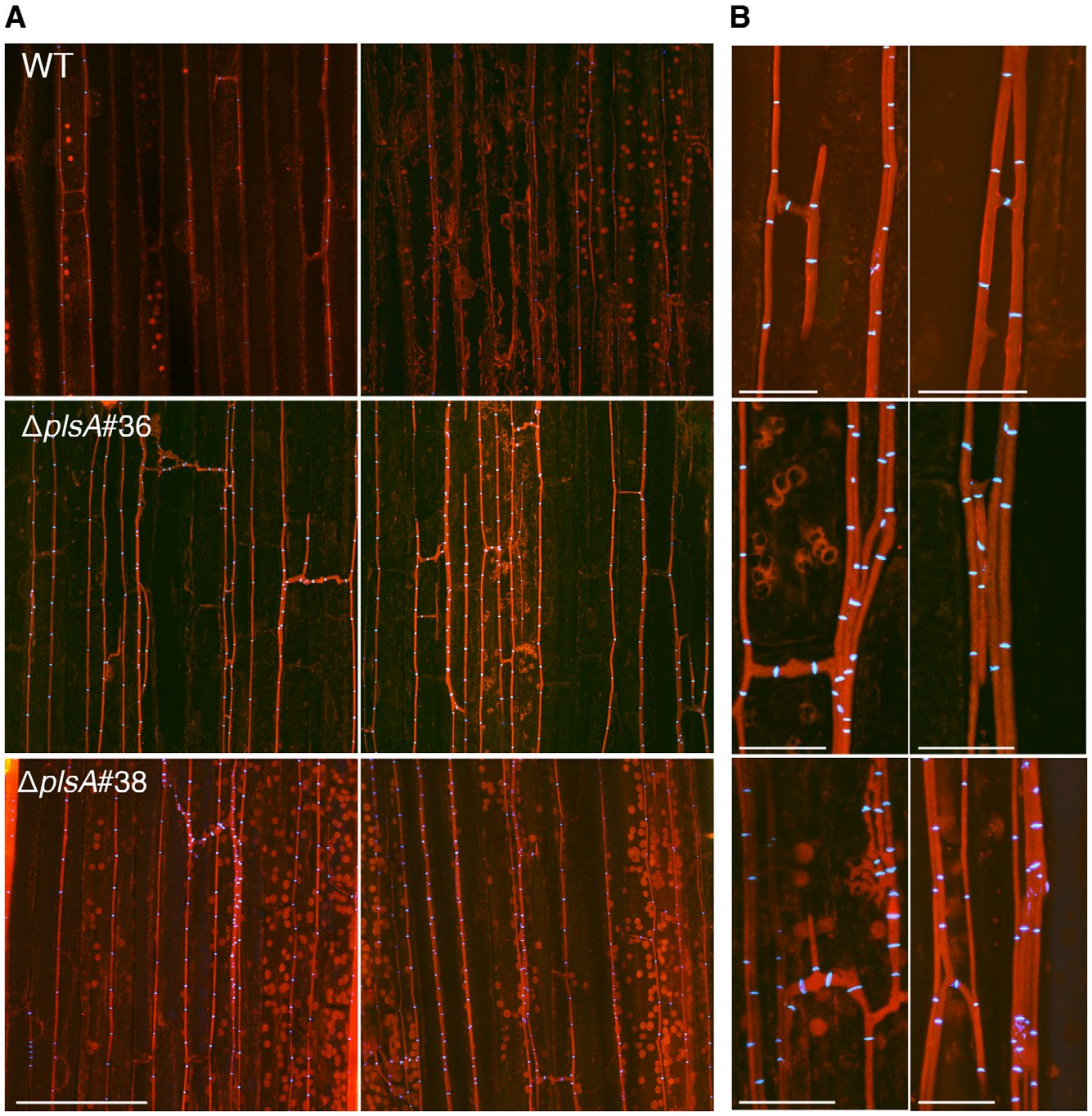
Confocal depth series images of *L. perenne* leaf sheaths infected with wild‐type and ∆*plsA* strains. (A) Two representative confocal *z*‐stack (7 μm) maximum intensity projections showing increased hyphal biomass during mutant colonization compared to wild‐type (WT). Bar = 100 μm. (B) Two representative higher magnification *z*‐stack maximum intensity projections showing multiple mutant hyphae in a common intercellular space. In comparison, wild type spaces contain 1‐2 hyphae. Bar = 20 μm. Samples were stained with aniline blue (detects β‐glucans, fluorescence labelled in red pseudocolour) and WGA‐AF488 (detects chitin, fluorescence shown in blue pseudocolour).

**Figure 5:**
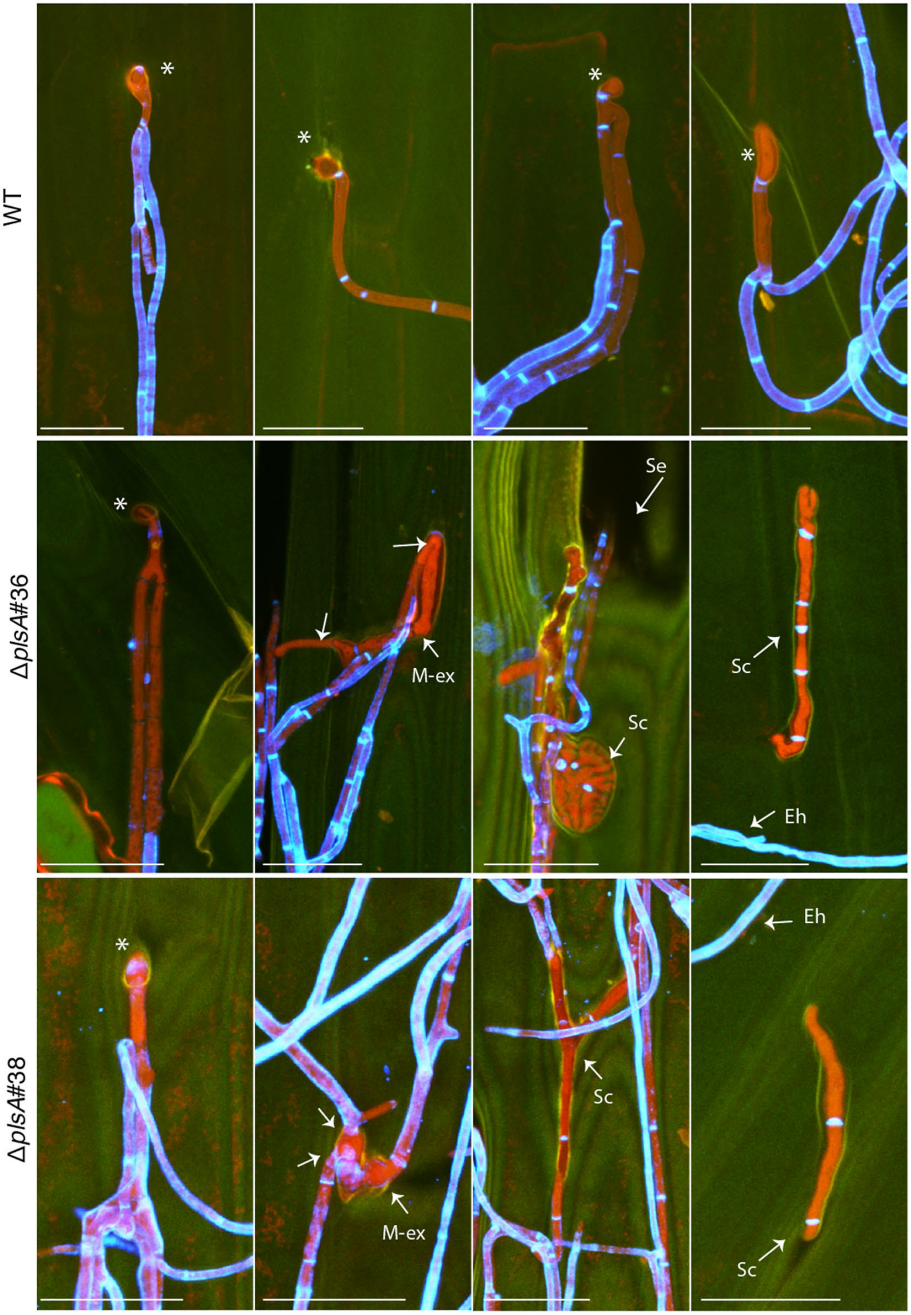
Confocal depth series images of wild‐type and mutant expressoria formation in *L. perenne* leaf sheaths. Wild‐type (WT) like expressoria swellings are indicated by asterisks (*). Epiphyllous hyphae (Eh) and hyphae exiting the host cuticle layer from malformed expressoria (M‐ex) and sub‐cuticular hyphae (Sc) are indicated. Samples were stained with aniline blue (detects β‐glucans, fluorescence shown in red pseudocolour) and WGA‐AF488 (detects chitin, fluorescence shown in blue pseudocolour). Fluorescence of the host cuticle layer is shown in green pseudocolour. Bar = 20 μm.

We next examined whether *plsA* is required for expressorium development and establishment of a mutualistic symbiotic interaction with *L. perenne* by inoculating wild‐type, Δ*plsA* and Δ*noxB* cultures into ryegrass seedlings and analysing host morphology at eight weeks post‐inoculation. Plants infected with the Δ*plsA* mutant had a reduced survival rate, a lower frequency of infection and mild host stunting (Fig. 2A–C; Table S1), whereas Δ*noxB* associations caused severe host stunting, as previously reported (Becker *et al.*, 2016). To determine the extent of host colonization and examine hyphal morphology *in planta*, host pseudostem tissue samples were harvested from wild‐type and Δ*plsA* associations and examined by transmission electron microscopy (TEM) and confocal laser scanning microscopy (CLSM). TEM analysis showed that Δ*plsA* hyphae colonize the host vascular bundles, whereas wild‐type hyphae were never observed in this region (Fig. 3A). TEM and CLSM analysis showed that mutant hyphae were more abundant in the intercellular spaces between host cells compared to wild‐type (Figs 3B,C and 4). Given expressorium formation requires the NoxA and NoxB complexes (Becker *et al.*, 2016), we examined the development of these structures. Wild‐type expressoria are characterized by formation of a swollen hyphal compartment that is delimited by a septum shortly after hyphal exit (Fig. 5). Hyphae then frequently branch and the cell wall is remodelled, as indicated by the abundant fluorescence of WGA‐AF488 indicative of chitin throughout the cell walls. A range of expressorium phenotypes were observed in Δ*plsA*‐infected *L. perenne* associations (Fig. 5), the most obvious being the presence of misshapen swellings with multiple hyphal exit points, or swellings, that were unable to penetrate the host cuticle layer, resulting in formation of subcuticular hyphae. Swellings that resembled normal wild‐type‐like expressoria were occasionally observed. Given NoxB plays a more crucial role than Pls1 in cellophane penetration (Fig. 1D), the ability of Δ*plsA* strains to form rare wild‐type‐like expressoria *in planta* (Fig. 4), a phenotype not observed for Δ*noxB*‐infected *L. perenne* associations (Becker *et al.*, 2016), was not surprising. To confirm the observed phenotypes were due to deletion of the *plsA* gene a wild‐type copy of the *plsA* gene was re‐introduced into the two Δ*plsA* mutant backgrounds. Plants infected with these transformants had the same asymptomatic host interaction phenotypes as wild‐type (Fig. S4). Collectively these results indicate that PlsA is required for proper expressorium differentiation and establishment of a mutualistic symbiotic interaction with *L. perenne*.

### The septin Sep3 assembles in a ring‐like structure in the expressorium

**Figure 6:**
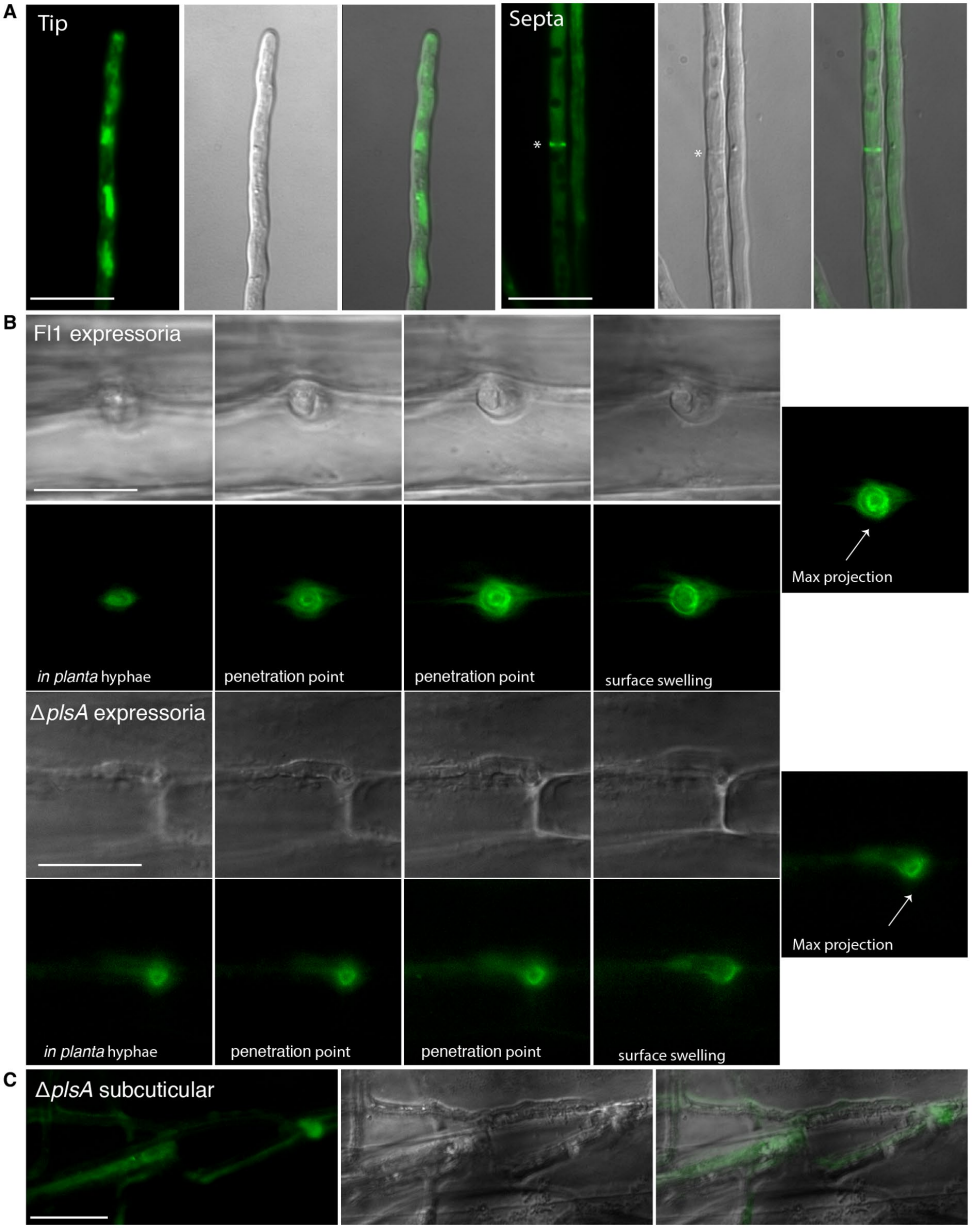
Sep3‐eGFP localization in wild‐type and ∆*plsA* cultures. (A) Wild‐type hyphae growing on 1.5% water agar for seven days expressing Sep3‐eGFP at hyphal tips and septa. Septa are marked with asterisks. Scale bar = 20 μm. (B) Incremental images showing Sep3‐eGFP ring formation within *in planta* hyphae as they exit the host cuticle by means of an expressorium. Scale bar = 20 μm. (C) Sep3 eGFP localization in mutant subcuticular hyphae. Subcuticular hyphae do not contain a septin ring. Scale bar = 20 μm.

In *M. oryzae*, four septins, Sep3, Sep4, Sep5 and Sep6, have been characterized (Dagdas *et al.*, 2012). Sep3 is essential for host penetration while Sep4, Sep5 and Sep6 are required for full virulence progression. Furthermore Sep3, Sep4 and Sep5 form a localized septin ring at the appressorium pore. Given the altered expressorium phenotypes observed for Δ*noxB* (Becker *et al.*, 2016) and Δ*plsA* strains, we examined how the *E. festucae* homologue of Sep3 is localized within the expressorium. Sep3 was chosen as it is essential for host penetration, rather than full virulence progression (Dagdas *et al.*, 2012). To examine this phenotype, a Sep3‐eGFP construct was transformed into wild‐type protoplasts and the transformants examined in culture and *in planta*. Similar to *M. oryzae* (Dagdas *et al.*, 2012), Sep3‐eGFP in hyphae of *E. festucae* in axenic culture localized to a patchy network in hyphal tips and to septa (Fig. 6A). *In planta,* Sep3‐eGFP localized to ring‐like structures within wild‐type expressoria at the points of host exit (Fig. 6B), results consistent with previous TEM analysis showing that septa occur at the base of the expressoria (Becker *et al.*, 2016). In subcuticular Δ*plsA* hyphae, which reached but never breached the host cuticle, Sep3‐eGFP was cytoplasmic and a polarized septin ring was never observed at the host cuticle (Fig. 6C). In comparison, a distinct Sep3‐eGFP ring was observed in Δ*plsA* hyphae which reached and breached the host cuticle by means of rare wild‐type‐like expressoria swellings (Fig. 6B). Collectively these results show that a Sep3 ring occurs within wild‐type‐like *E. festucae* expressoria and that this ring is absent in subcuticular Δ*plsA* hyphae, which fail to polarize correctly and differentiate into expressoria when they reach the host cuticle.

## Discussion

In many plant–fungal interactions, ROS production from the catalytic activity of the NoxA and NoxB complexes is important for successful host penetration and colonization (Scott, 2015; Tudzynski *et al.*, 2012). Here we have shown that the homologue of Pls1, PlsA, an adaptor protein for the NoxB complex (Zhao *et al.*, 2016), is required for *E. festucae* expressorium differentiation and for establishment of a mutualistic symbiotic interaction with *L. perenne*. Imaging of Sep3‐eGFP localization *in planta* showed that wild‐type *E. festucae* expressoria contain a Sep3‐eGFP ring that is absent in subcuticular hyphae of the Δ*plsA* mutant.

In filamentous fungi, ROS have an important role in the establishment and maintenance of hyphal polarity, co‐ordinated hyphal growth, cell–cell communication and the development of sexual fruiting bodies (Herzog *et al.*, 2015; Scott, 2015; Tudzynski *et al.*, 2012). In many fungi, components of the NoxA complex are required for fruiting body formation and cell–cell fusion, whereas NoxB/Nox2 components are dispensable for these processes (Cano‐Domínguez *et al.*, 2008; Dirschnabe *et al.*, 2014; Malagnac *et al.*, 2004; Roca *et al.*, 2012; Tong *et al.*, 2014). In *P. anserina,* PaNox2 and PaPls1 are essential for hyphal re‐orientation/polarization towards cellophane membranes, whereas PaNox1 is dispensable (Brun *et al.*, 2009). Thus, in axenic culture there is a clear separation between Nox signalling, where NoxA/Nox1 contributes to cell–cell fusion and fruiting body formation, and Nox2 contributes to hyphal re‐polarization on cellophane. Unlike many other fungi that form fruiting bodies in culture, the sexual cycle of *E. festucae* has only been studied *in planta* (Schardl, 1996). Previously, defects within either *E. festucae* NoxA or NoxB signalling in culture have only been distinguished using cell–cell fusion assays. Here we developed a second assay using cellophane membranes that further differentiates *E. festucae* NoxA and NoxB defects. In axenic culture, *E. festucae* Δ*noxA*, Δ*noxR*, Δ*racA* and Δ*bemA* hyphae are defective in cell–cell fusion, whereas Δ*noxB* hyphae are indistinguishable from wild‐type (Kayano *et al.*, 2013). Here we have shown that similar to *E. festucae* NoxB (Kayano *et al.*, 2013), PlsA is not required for NoxA‐mediated hyphal fusion in culture. Furthermore, NoxB is essential, Pls1 is partially required, and NoxA and NoxR are dispensable for hyphae to breach cellophane. These results are consistent with Pls1 being an adaptor protein for NoxB/Nox2, which contributes to NoxB signalling (Scott, 2015; Tudzynski *et al.*, 2012; Zhao *et al.*, 2016). In *P. anserina,* PaNox2 and PaPls1 mutant hyphae do not re‐orientation/re‐polarize towards cellophane membranes, whereas PaNox1 hyphae reorient and establish contacts more prominently than wild‐type and possess enhanced cellulolytic capacity, thereby allowing PaNox1 hyphae to efficiently degrade cellulose without penetration (Brun *et al.*, 2009). PaNox1 and PaPls1 are also required to grow hyphae out of melanized structures, whether they are appressoria or ascospores (Lambou *et al.*, 2008). *E. festucae* Δ*noxA* hyphae appear to have a similar cellophane penetration phenotype, given they are able to breach cellophane and many genes involved in cell wall degradation are upregulated in the Δ*noxA* mutant *in planta* compared to the wild‐type strain (Eaton *et al.*, 2015). Interestingly, *E. festucae* Δ*noxB* and Δ*plsA* phenotypes are not identical. NoxB is more important than Pls1 for hyphae to breach cellophane.

In *B. cinerea*, Nox2 is required for the differentiation of appressorium‐like structures and host penetration, whereas Nox1 is required for virulent growth *in planta* (Segmüller *et al.*, 2008). In *M. oryzae*, the F‐actin cytoskeleton, septins Sep3, Sep4 and Sep5, and the exocyst components Sec3, Sec5, Sec6, Sec8, Sec15, Exo70 and Exo84 are recruited to the base of the appressorium in a ring‐like structure. NoxR and Nox2 are required for Sep5‐eGFP and Sec6‐eGFP recruitment whereas Nox1 is dispensable, suggesting that septin‐dependent host penetration requires the Nox2 rather than the Nox1 complex (Dagdas *et al.*, 2012; Gupta *et al.*, 2015; Ryder *et al.*, 2013). Similar to *M. oryzae*, a Sep4‐eGFP ring forms in *B. cinerea* appressoria (Feng *et al.*, 2017), and an exocyst VdSec8‐eGFP/VdExo70‐eGFP and a NoxB‐dependent Sep5‐eGFP ring form in *V. dahliae* hyphopodia (Zhao *et al.*, 2016, 2017). Furthermore, VdPls1 and VdNoxB localize to *V. dahliae* hyphopodia penetration points (Zhao *et al.*, 2016). Collectively these studies indicate that in many fungi a septin ring forms within developing host penetration structures and there is a clear separation between Nox2 and Nox1 signalling *in planta*. Nox2 is required for the recruitment of septin and exocyst components during initial host penetration, whereas Nox1 is dispensable. In the initial stages of *E. festucae*–host colonization, endophytic hyphae grow by tip growth within the true stem, then switch to intercalary growth in the sheath and blade tissues to establish a restricted network of hyphae within the host (Eaton *et al.*, 2012). Hyphal tip contact with the host cuticle triggers formation of a swollen hyphal compartment and differentiation of an expressorium and localized host penetration (Becker *et al.*, 2016). Deletions of *noxA*, *noxB*, *noxR*, *racA* and *bemA* result in prolific hyphal growth, increased hyphal biomass and stunting of the host plant. Other phenotypes of these mutants *in planta* include the formation of convoluted hyphal structures with multiple hyphae packed in between host cells and increased colonization of the host vascular tissues (Becker *et al.*, 2016; Takemoto *et al.*, 2006, 2011; Tanaka *et al.*, 2006, 2008). Furthermore, NoxA, NoxB and NoxR are essential for expressorium formation *in planta* (Becker *et al.*, 2016). Here we have shown that in comparison to Δ*noxB*, the Δ*plsA* hyphae cause similar but less severe host interaction defects. Although Δ*plsA* strains can occasionally form wild‐type‐like expressoria, hyphal tips predominantly fail to polarize or differentiate into expressoria and instead form a subcuticular hyphal network similar to that seen in Δ*noxA*, Δ*noxB*, Δ*noxR*, Δ*symB,* Δ*symC* and Δ*mobC* associations (Becker *et al.*, 2016; Green *et al.*, 2016, 2017). Similar to plant‐pathogenic fungi that contain a septin ring in appressoria (Dagdas *et al.*, 2012; Feng *et al.*, 2017; Gupta *et al.*, 2015; Ryder *et al.*, 2013; Zhao *et al.*, 2016; Zhou *et al.*, 2017), a Sep3‐eGFP ring forms within *E. festucae* wild‐type‐like expressoria that is absent in subcuticular Δ*plsA* hyphae. Collectively our results show that NoxB has a more important role than Pls1 in expressorium development and in the establishment and maintenance of a mutualistic symbiotic interaction with the grass host. Furthermore, PlsA contributes to, but is not essential for, NoxB‐mediated recruitment and/or maintenance of the septin ring. In contrast, *V. dahliae* (Zhao *et al.*, 2016), *M. oryzae* (Clergeot *et al.*, 2001; Egan *et al.*, 2007), the *B. cinerea* highly virulent T4 strain (Gourgues *et al.*, 2004) and *C. lindemuthianum* (Veneault‐Fourrey *et al.*, 2005), *pls1* and *nox2* mutants are identical in their inability to penetrate host tissues. In the mutualistic endophyte *E. festucae*, PlsA and NoxB appear to have adapted slightly different regulatory roles and signalling requirements, presumably due to different life‐cycle, host‐colonization or host‐penetration selection pressures. In contrast to Nox2/NoxB, which comprises the catalytic component of the complex, Pls1/PlsA has been proposed to be a scaffolding protein for tethering or recruiting other signalling components (Scott, 2015). Unlike *E. festucae* NoxA cytosolic components, NoxB cytosolic components are largely unknown. While NoxB, Pls1, NoxR and Bem1 function together on the outside of the ER in *B. cinerea*, NoxB has been proposed to function alone within the ER, emphasising the dynamic nature of Nox complex assembly depending on the stage of development and the cellular localization (Marschall *et al.*, 2016). In *M. oryzae* the Nox complex regulatory protein NoxR, the Nox1 adaptor protein NoxD and the Nox2 adaptor protein Pls1 are essential for septin ring formation in the appressorium, whereas Nox1 is dispensable (Galhano *et al.*, 2017; Ryder *et al.*, 2013). These findings suggest that Pls1 functions together with NoxD and Nox2 in regulating the formation of polarization structures (Galhano *et al.*, 2017). Interestingly, *E. festucae* Δ*plsA* defects are less severe than Δ*noxB* defects both on cellophane and *in planta*. Two hypotheses to explain these findings are (1) the catalytic component NoxB acts alone in certain differentiation processes as proposed for *B. cinerea* (Marschall *et al.*, 2016) or (2) an alternative signalling mechanism or scaffold protein facilitates NoxB activation and hyphal polarisation in the absence of PlsA.

Although ROS play a major role in regulating *E. festucae* growth *in planta*, the NoxA and NoxB complexes are not the only signalling pathways required for controlling the establishment and maintenance of a mutualistic symbiotic association (Scott *et al.*, 2018). A siderophore for maintaining iron homeostasis (Johnson *et al.*, 2013), the transcription factor ProA (Green *et al.*, 2017; Tanaka *et al.*, 2013), the stress‐activated and cell wall integrity mitogen‐activated protein kinase (MAPK) signalling pathways (Becker *et al.*, 2015; Eaton *et al.*, 2010), the STRIPAK complex (Green *et al.*, 2016), cAMP/PKA (Voisey *et al.*, 2016) and calcineurin (Mitic *et al.*, 2018) are all required for maintaining the symbiosis and cause similar host‐association defects when disrupted. Given the similarities between mutant phenotypes, the molecular mechanisms which facilitate gene regulation and cross talk between the Nox complex components and the different signalling pathways in *E. festucae*–host associations are of considerable interest and still relatively poorly described. In *S. macrospora* the transcription factor PRO1 has been shown to bind to the promoter regions of *nox1*, *pro41 (noxD)*, *pls1* and *nox2* during fruiting body development, suggesting PRO1 regulates the expression of Nox complex genes (Steffens *et al.*, 2016). In *E. festucae*, however, although ProA is required for the symbiotic interaction (Tanaka *et al.*, 2013) and the promoter region of *plsA* contains a putative ProA binding site, *plsA* is not differentially expressed in mutant Δ*proA*–host associations (Eaton *et al.*, 2015), suggesting ProA does not regulate *plsA* expression in vegetative tissue. In *P. anserina*, PaNox1 promotes nuclear translocation of the cell‐wall integrity pathway MAPK PaMpk1 (Kicka *et al.*, 2006) and in *B. cinerea* the IQGAP homologue BcIqg1 interacts with the Nox complex, MAP kinase and calcium signalling proteins to regulate virulence and development (Marschall and Tudzynski, 2016). These results collectively suggest there is cross talk between Nox, MAPK and calcium signalling pathways. Interestingly, both the cell‐wall integrity and stress‐activated MAP kinase pathways (Becker *et al.*, 2015; Eaton *et al.*, 2010) and calcineurin (Mitic *et al.*, 2018) signalling pathways are required for the *E. festucae*–*L. perenne* symbiotic interaction. Whether similar cross‐talk between these pathways and the Nox complex occurs in *E. festucae* remains to be determined.

In summary, we have shown that the *E. festucae* tetraspanin PlsA contributes to expressorium differentiation and mutualistic colonization of the grass host *L. perenne*. Furthermore, analysis of Sep3‐eGFP distribution indicates that a septin‐based ring forms in expressoria that is absent in subcuticular Δ*plsA* hyphae. We have also developed a cellophane penetration assay for *Epichloë* that will help distinguish between NoxB and NoxA defects in culture. These findings provide new insights into how the proposed Nox2 complex adaptor protein PlsA affects *E. festucae*–host associations and how the septin Sep3 is organized in expressoria. Whether or not the Nox2 complex facilitates the recruitment of septins and/or exocyst components within *E. festucae* expressoria and what other signalling pathways are required for this differentiation process are key questions for the future.

## Experimental Procedures

### Growth conditions and endophyte inoculations

*Saccharomyces cerevisiae* cultures were grown in yeast extract peptone dextrose (YPD) media or on YPD agar plates (Colot *et al.*, 2006). *Escherichia coli* cultures were grown in Lysogeny Broth (LB) broth or on LB agar plates supplemented with 100 μg/mL ampicillin (Miller, 1972). *E. festucae* cultures were grown on 2.4% (w/v) potato dextrose (PD) agar plates or in PD broth (Moon *et al.*, 1999, 2000). Endophyte‐free seedlings of perennial ryegrass (*Lolium perenne* cv. Samson) were inoculated as previously described (Latch and Christensen, 1985). Plants were grown at 22°C with a photoperiod of 16 h of light (~100 μmol/m^2^ per second) in an environmentally controlled growth room and tested for the presence of the endophyte by immunoblotting (Tanaka *et al.*, 2005).

### DNA isolation, PCR and sequencing

*S. cerevisiae* plasmid DNA was extracted as previously described (Colot *et al.*, 2006). *E. coli* plasmid DNA was extracted using the High Pure Plasmid Isolation Kit (Roche, Indianapolis, IN, USA). Fungal DNA was extracted as previously described (Byrd *et al.*, 1990). Cloning and deletion mutant screening PCR reactions were performed using Phusion® High Fidelity DNA Polymerase (Thermo Scientific, Waltham, MA, USA) and Taq DNA polymerase (Roche), respectively. Sequencing reactions were performed using the Big‐DyeTM Terminator Version 3.1 Ready Reaction Cycle Sequencing Kit (Applied BioSystems, Carlsbad, California, USA) and separated using an ABI3730 genetic analyser (Applied Bio Systems). Sequence data were assembled and analysed using MacVector sequence assembly software, version 12.0.5.

### Construct preparation and transformations

Biological materials and primers can be found in Tables S2 and S3. The *plsA* replacement construct (pCE60) was prepared by yeast recombinational cloning (Gietz and Woods, 2002). *Eco*RI/*Xho*I restriction enzyme linearized pRS426 vector backbone was recombined with a 1616 bp 5′ *plsA* flank (primers pRS426‐plsA‐F/plsA‐hph‐R, genomic *E. festucae* wild‐type DNA), a 702 bp 3′ *plsA* flank (primers hph‐plsA‐F/plsA‐pRS426‐R, genomic wild‐type DNA) and a 1.4 kb P*trpC‐hph* cassette (primers hphF/R, plasmid pSF15.15 DNA). The *plsA* complementation construct (pKG34) was prepared by Gibson Assembly (Gibson *et al.*, 2009). A 2.6‐kb ampicillin resistance vector (primers pRS426_F/pRS426_R, plasmid pAN7‐1 DNA) was recombined with a 3 kb fragment containing the native *plsA* gene (primers KG150/151, genomic wild‐type DNA). The *E. festucae* homologue of Sep3 (EfM3.035910) was identified using a tBLASTn search of the *E. festucae* wild‐type (E894) genome sequence using *M. oryzae* Sep3 (MGG_01521) as the query sequence. The Sep3‐3xGA‐eGFP construct (pKG36) was prepared by Gibson Assembly (Gibson *et al.*, 2009) by recombining the *tefA* promoter, 3xGA‐eGFP, TrpC terminator, ampicillin and hygromycin resistance cassettes (primers gfpF/ptefR, plasmid pPN94 DNA) with the *sep3* gene sequence (primers KG158/155, genomic wild‐type DNA). Recombined plasmids were transformed into electrocompetent DH5α *E. coli*, transformants selected on ampicillin, and plasmids purified from *E. coli* and confirmed to be error‐free by sequencing. *E. festucae* protoplasts were prepared (Young *et al.*, 2005) and transformed with 2–3 μg of target DNA (Itoh *et al.*, 1994) using hygromycin (150 μg/mL) or geneticin (200 μg/mL) selection. The *plsA* replacement fragment was amplified from pCE60 plasmid DNA using the primers pRS426‐plsA‐F and plsA‐pRS426‐R, and transformed into *E. festucae* wild‐type protoplasts using hygromycin selection. The *plsA* complementation construct pKG34 was co‐transformed with pII99 into Δ*plsA* protoplasts using geneticin selection. The Sep3‐eGFP construct pKG36 was transformed into wild‐type protoplasts using hygromycin selection. Transformants were nuclear purified by three rounds of sub‐culturing.

### Southern analysis

*E. festucae* genomic digests, separated by agarose gel electrophoresis, were transferred to positively charged nylon membranes (Roche) (Southern, 1975) and fixed by UV light cross‐linking in a Cex‐800 UV light cross‐linker (Ultra‐Lum, Claremont, California, USA) at 254 nm for 2 min. Digoxigenin‐dUTP (DIG) labelling and hybridization of the *plsA* DNA probe and nitroblue tetrazolium chloride and 5‐bromo‐4‐chloro‐3‐indolyl‐phosphate (NBT/BCIP) visualization were performed as per the manufacturer’s instructions using the DIG High Prime DNA Labelling and Detection Starter Kit I (Roche).

### Microscopy

*E. festucae* cultures were grown on glass slides overlaid with 1.5% (w/v) water agar. Culture morphology and growth, and Sep3‐eGFP localization in culture and *in planta*, were analysed using an Olympus IX83 inverted fluorescence microscope, using setups for both Differential interference contrast (DIC) and standard GFP filters. Infected pseudostem tissue for CLSM was stained and images captured as previously described (Becker *et al.*, 2018) using a Leica SP5 DM6000B confocal microscope (excitation wavelengths of 488 nm and 561 nm, emitted light was collected from 498–551, 593–625 and 661–796 nm, respectively, using either a 40 × or 63 × oil immersion objective, Numerical Aperture (NA) = 1.4) (Leica Microsystems, Wetzlar, Germany). Pseudostem sections for TEM analysis were fixed in 3% glutaraldehyde and 2% formaldehyde in 0.1 M phosphate buffer, pH 7.2 as previously described (Spiers and Hopcroft, 1993). Images were acquired using a Philips CM10 TEM and a SIS Morada digital camera.

### Bioinformatics

The *E. festucae* wild‐type (E894) genome is available at http://csbio-l.csr.uky.edu/ef894-2011/ (Schardl *et al.*, 2013a). Pls1 and Sep3 protein sequences from several fungal species as listed in Fig. S1A were obtained from NCBI (http://www.ncbi.nlm.nih.gov/). ClustalW pairwise protein sequence alignment (Thompson *et al.*, 1994) was performed using MacVector 12.0.5 software. Domain analysis was performed using InterProScan (v. 5) (Quevillon *et al.*, 2005; Zdobnov and Apweiler, 2001) and TMHMM (v. 2.0c) (Krogh *et al.*, 2001; Sonnhammer *et al.*, 1998). ProA binding sites were identified manually using the *E. festucae* ProA binding domain described previously (Tanaka *et al.*, 2013).

## Supporting information


**Fig. S1** *Epichloë festucae* *plsA* gene structure, encoded protein domain structure and amino acid sequence alignment. (A) Gene structure of *E. festucae* *plsA* containing three exons of 402, 166 and 107 base pairs (bp), and two introns of 90 and 89 bp. (B) Protein domain structure of *E. festucae* PlsA. TMHMM software (v. 2.0c) (Krogh* et al.*, 2001; Sonnhammer* et al.*, 1998) predicts four trans membrane domains (TMD, blue). InterProScan (v. 5) (Quevillon* et al.*, 2005; Zdobnov and Apweiler, 2001) predicts a tetraspanin EC2 domain between TMD3 and TMD4 (yellow). (C) Multiple amino acid (aa) sequence alignment (ClustalW) of PlsA homologues from Ef, *E. festucae* EfM3.019170; *Nc*, *Neurospora crassa* NCU07432 (XM_959526.2); *Sm*, *Sordaria macrospora* SMAC_05218 (XM_003346971.1); *Pa*, *Podospora anserina* Pa_1_19270 (XM_001907355.1); *Mo*, *Magnaporthe oryzae* MGG_12594 (XM_003720928.1) and *Fg*, *Fusarium graminearum* FGSG_08695 (XM_011321741.1). TMDs and the EC2 domain are coloured as in B. Conserved cysteine residues (red) within the EC2 domain and predicted *N* glycosylated residues (green) are indicated.

**Fig. S2** Identification of ProA binding site in the *plsA* promoter of various *Epichloë* spp. Multiple sequence alignment of 1 kb *plsA *promoter regions from several *Epichloë* species including *E. baconii, E. bromicola, E. elymi*, *E. typhina*, *E. festucae *Fl1 and *E. amarillans*. The conserved predicted ProA binding site is shaded in red and ATG start site in green.

**Fig. S3** *plsA* replacement strategy and Southern analysis of ∆*plsA *strains. (A) Physical maps of strain Fl1 wild type *plsA* and mutant ∆*plsA* loci and linear inserts of *plsA *replacement, pCE60 (top), and complementation, pKG34 (bottom) constructs. Regions of recombination are indicated by grey shading. Primer pairs used to amplify genomic 5ʹ and 3ʹ flanking regions and the hygromycin (*hph*) resistance cassette and primer pairs used for ∆*plsA *PCR screening are shown. *Eco*RI restriction enzyme sites used for Southern analysis are as shown. Bar   2 kb. (B) NBT BCIP stained Southern blot of *Eco*RI genomic DNA digests (1.5 μg) probed with (DIG) 11 dUTP labelled linear pCE60 PCR fragment (primers pRS426 plsA F plsA pRS426 R). Fragments of the expected size for wild type (6.6 kb) and  clean  (7.3 kb) integration are as shown.

**Fig. S4** Complementation of ∆*plsA* defects *in planta*. (A) Host morphology in wild type, ∆*plsA*, ∆*noxB* and ∆*plsA* *plsA* (C1 3) associations at seven weeks post planting. (B) Height of the tallest infected tiller (wild type, ∆*plsA* and ∆*plsA/plsA *associations *n*   14 18; ∆*noxB *associations *n*   3). An asterisk indicates significant differences from wild type (*P*   0.05), as determined by one way ANOVA test.

**Table S1** Survival and infection rates of wild type, mutant and complemented strains.

**Table S2** Biological material.

**Table S3** Primers used in this study.
