# Supplementary material for: A homologue of the fungal tetraspanin Pls1 is required for Epichloë festucae expressorium formation and establishment of a mutualistic interaction with Lolium perenne
Source: Mol Plant Pathol. 2019 Apr 22;20(7):961–75. doi: 10.1111/mpp.12805 (PMC6589725; doi:10.1111/mpp.12805)
Supplement: Supplementary file 1 — Fig. S1 Epichloë festucae plsA gene structure, encoded protein domain structure and amino acid sequence alignment. (A) Gene structure of E. festucae plsA containing three exons of 402, 166 and 107 base pairs (bp), and two introns of 90 and 89 bp. (B) Protein domain structure of E. festucae PlsA. TMHMM software (v. 2.0c) (Krogh et al., 2001; Sonnhammer et al., 1998) predicts four trans membrane domains (TMD, blue). InterProScan (v. 5) (Quevillon et al., 2005; Zdobnov and Apweiler, 2001) predicts a tetraspanin EC2 domain between TMD3 and TMD4 (yellow). (C) Multiple amino acid (aa) sequence alignment (ClustalW) of PlsA homologues from Ef, E. festucae EfM3.019170; Nc, Neurospora crassa NCU07432 (XM_959526.2); Sm, Sordaria macrospora SMAC_05218 (XM_003346971.1); Pa, Podospora anserina Pa_1_19270 (XM_001907355.1); Mo, Magnaporthe oryzae MGG_12594 (XM_003720928.1) and Fg, Fusarium graminearum FGSG_08695 (XM_011321741.1). TMDs and the EC2 domain are coloured as in B. Conserved cysteine residues (red) within the EC2 domain and predicted N glycosylated residues (green) are indicated. [file MPP-20-961-s001.docx]

**
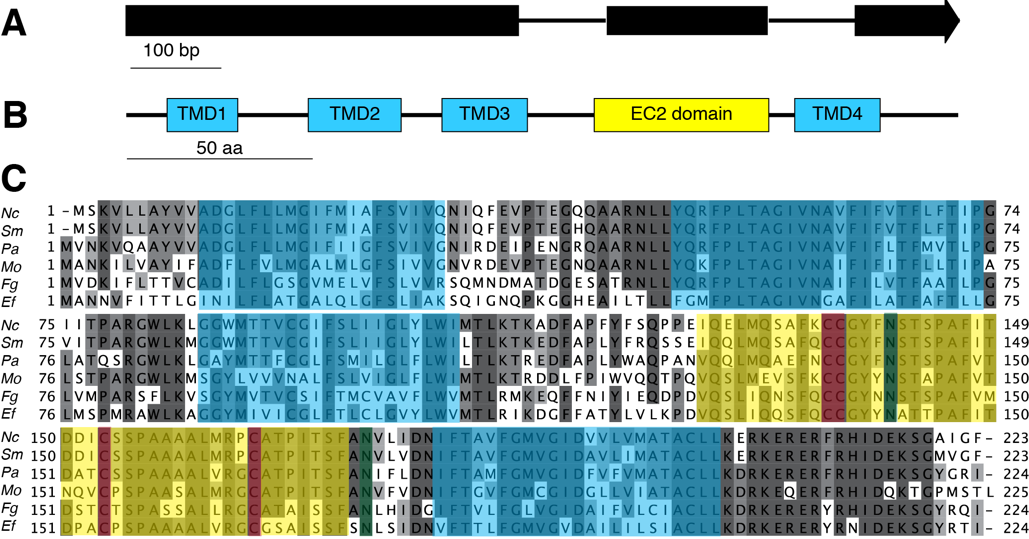
**

**Fig. S1.** *Epichloë festucae* *plsA* gene structure, encoded protein domain structure and amino acid sequence alignment. (A) Gene structure of *E. festucae* *plsA* containing three exons of 402, 166 and 107 base pairs (bp), and two introns of 90 and 89 bp. (B) Protein domain structure of *E. festucae* PlsA. TMHMM software (v. 2.0c) (Sonnhammer *et al.*, 1998, Krogh *et al.*, 2001) predicts four trans-membrane domains (TMD, blue). InterProScan (v. 5) (Zdobnov & Apweiler, 2001, Quevillon *et al.*, 2005) predicts a tetraspanin EC2 domain between TMD3 and TMD4 (yellow). (C**)** Multiple amino acid (aa) sequence alignment (ClustalW) of PlsA homologues from Ef, *Epichloë festucae* EfM3.019170; *Nc*, *Neurospora crassa* NCU07432 (XM_959526.2); *Sm*, *Sordaria macrospora* SMAC_05218 (XM_003346971.1); *Pa*, *Podospora anserina* Pa_1_19270 (XM_001907355.1); *Mo*, *Magnaporthe oryzae* MGG_12594 (XM_003720928.1) and *Fg*, *Fusarium graminearum* FGSG_08695 (XM_011321741.1). TMDs and the EC2 domain are coloured as in B. Conserved cysteine residues (red) within the EC2 domain and predicted N-glycosylated residues (green) are indicated.

**REFFERENCES**

**Krogh, A., Larsson, B., von Heijne, G. and Sonnhammer, E. L.** (2001) Predicting transmembrane protein topology with a hidden Markov model: application to complete genomes. *J Mol Biol,* **305,** 567–580.

**Quevillon, E., Silventoinen, V., Pillai, S., Harte, N., Mulder, N. and Apweiler, R.** (2005) InterProScan: protein domains identifier. *Nucleic Acids Res,* **33,** W116–W120.

**Sonnhammer, E. L., von Heijne, G. and Krogh, A.** (1998) A hidden Markov model for predicting transmembrane helices in protein sequences. In: *International Conference on Intelligent Systems for Molecular Biology.* ISMB, pp. 175-182.

**Zdobnov, E. M. and Apweiler, R.** (2001) InterProScan–an integration platform for the signature-recognition methods in InterPro. *Bioinformatics,* **17,** 847-848.
