# Supplementary material for: A homologue of the fungal tetraspanin Pls1 is required for Epichloë festucae expressorium formation and establishment of a mutualistic interaction with Lolium perenne
Source: Mol Plant Pathol. 2019 Apr 22;20(7):961–75. doi: 10.1111/mpp.12805 (PMC6589725; doi:10.1111/mpp.12805)
Supplement: Supplementary file 2 — Fig. S2 Identification of ProA binding site in the plsA promoter of various Epichloë spp. Multiple sequence alignment of 1 kb plsA promoter regions from several Epichloë species including E. baconii, E. bromicola, E. elymi, E. typhina, E. festucae Fl1 and E. amarillans. The conserved predicted ProA binding site is shaded in red and ATG start site in green. [file MPP-20-961-s002.docx]

**
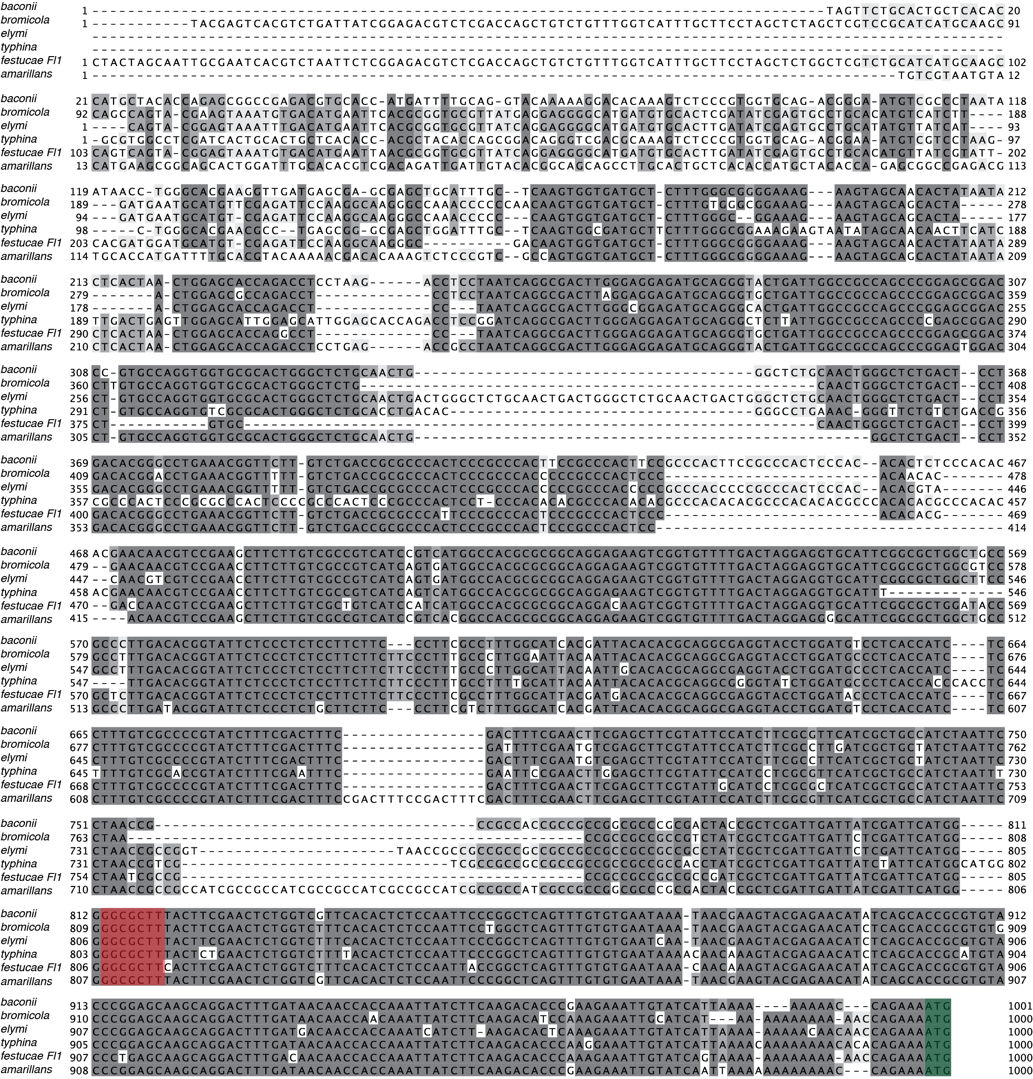
**

**Fig. S2.** Identification of ProA binding site in the *plsA* promoter of various *Epichloë* *spp*. Multiple sequence alignment of 1 kb *plsA* promoter regions from several *Epichloë* species including *E. baconii, E. bromicola, E. elymi*, *E. typhina*, *E. festucae* Fl1 and *E. amarillans*. The conserved predicted ProA binding site is shaded in red and ATG start site in green.
