# Supplementary material for: A homologue of the fungal tetraspanin Pls1 is required for Epichloë festucae expressorium formation and establishment of a mutualistic interaction with Lolium perenne
Source: Mol Plant Pathol. 2019 Apr 22;20(7):961–75. doi: 10.1111/mpp.12805 (PMC6589725; doi:10.1111/mpp.12805)
Supplement: Supplementary file 3 — Fig. S3 plsA replacement strategy and Southern analysis of ∆plsA strains. (A) Physical maps of strain Fl1 wild type plsA and mutant ∆plsA loci and linear inserts of plsA replacement, pCE60 (top), and complementation, pKG34 (bottom) constructs. Regions of recombination are indicated by grey shading. Primer pairs used to amplify genomic 5ʹ and 3ʹ flanking regions and the hygromycin (hph) resistance cassette and primer pairs used for ∆plsA PCR screening are shown. EcoRI restriction enzyme sites used for Southern analysis are as shown. Bar 2 kb. (B) NBT BCIP stained Southern blot of EcoRI genomic DNA digests (1.5 μg) probed with (DIG) 11 dUTP labelled linear pCE60 PCR fragment (primers pRS426 plsA F plsA pRS426 R). Fragments of the expected size for wild type (6.6 kb) and clean (7.3 kb) integration are as shown. [file MPP-20-961-s003.docx]

**
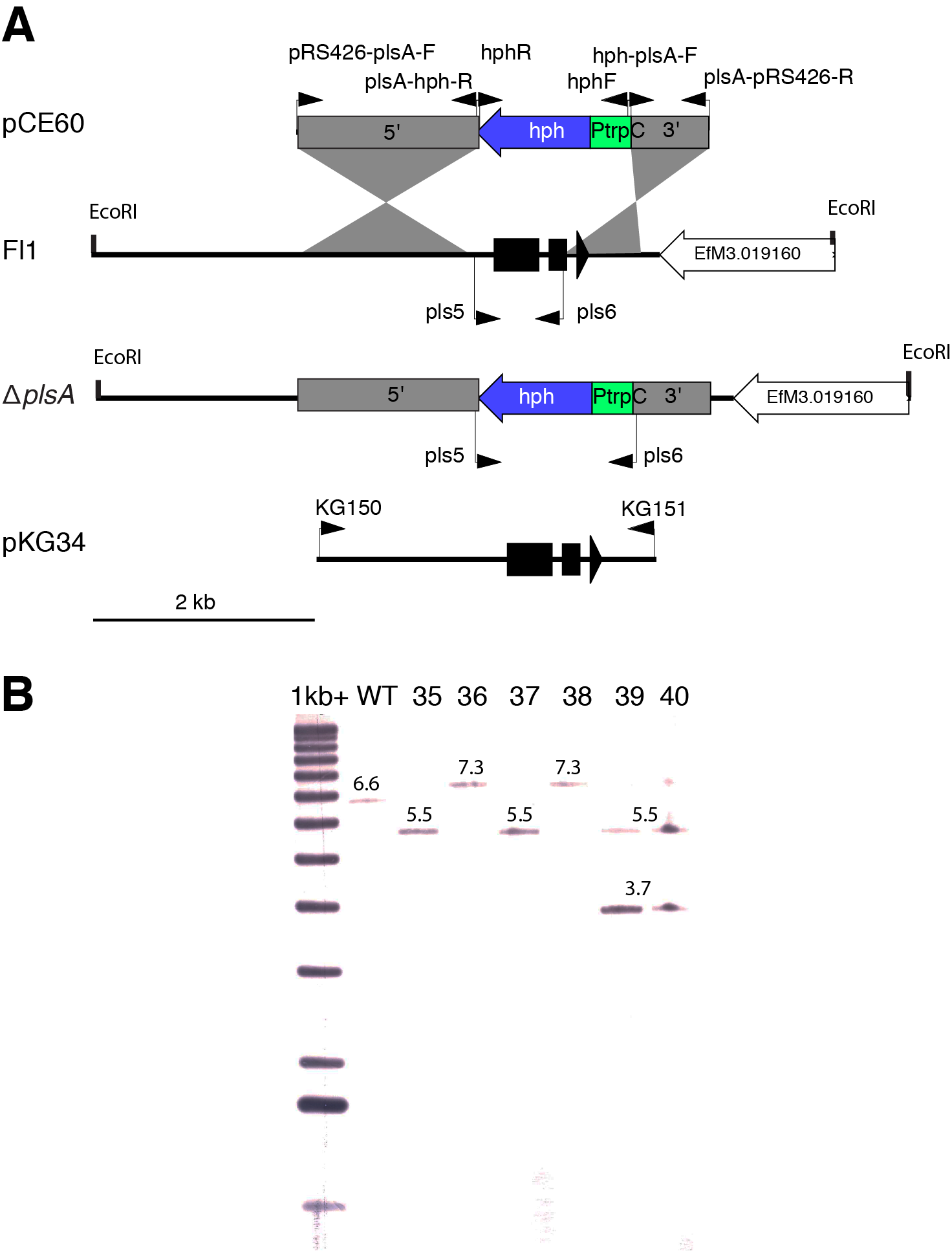
**

**Fig. S3.** *plsA* replacement strategy and Southern analysis of ∆*plsA* strains. (A**)** Physical maps of strain Fl1 wild type *plsA* and mutant ∆*plsA* loci and linear inserts of *plsA* replacement, pCE60 (top), and complementation, pKG34 (bottom) constructs. Regions of recombination are indicated by grey shading. Primer pairs used to amplify genomic 5’ and 3’ flanking regions and the hygromycin (*hph*) resistance cassette and primer pairs used for ∆*plsA* PCR screening are shown. *Eco*RI restriction enzyme sites used for Southern analysis are as shown. Bar = 2 kb. (B**)** NBT/BCIP stained Southern blot of *Eco*RI genomic DNA digests (1.5 μg) probed with (DIG)-11-dUTP labelled linear pCE60 PCR fragment (primers pRS426-plsA-F/plsA-pRS426-R). Fragments of the expected size for wild-type (6.6 kb) and ‘clean’ (7.3 kb) integration are as shown.
