# Supplementary material for: A homologue of the fungal tetraspanin Pls1 is required for Epichloë festucae expressorium formation and establishment of a mutualistic interaction with Lolium perenne
Source: Mol Plant Pathol. 2019 Apr 22;20(7):961–75. doi: 10.1111/mpp.12805 (PMC6589725; doi:10.1111/mpp.12805)
Supplement: Supplementary file 4 — Fig. S4 Complementation of ∆plsA defects in planta. (A) Host morphology in wild type, ∆plsA, ∆noxB and ∆plsA plsA (C1 3) associations at seven weeks post planting. (B) Height of the tallest infected tiller (wild type, ∆plsA and ∆plsA/plsA associations n 14 18; ∆noxB associations n 3). An asterisk indicates significant differences from wild type (P 0.05), as determined by one way ANOVA test. [file MPP-20-961-s004.docx]

**
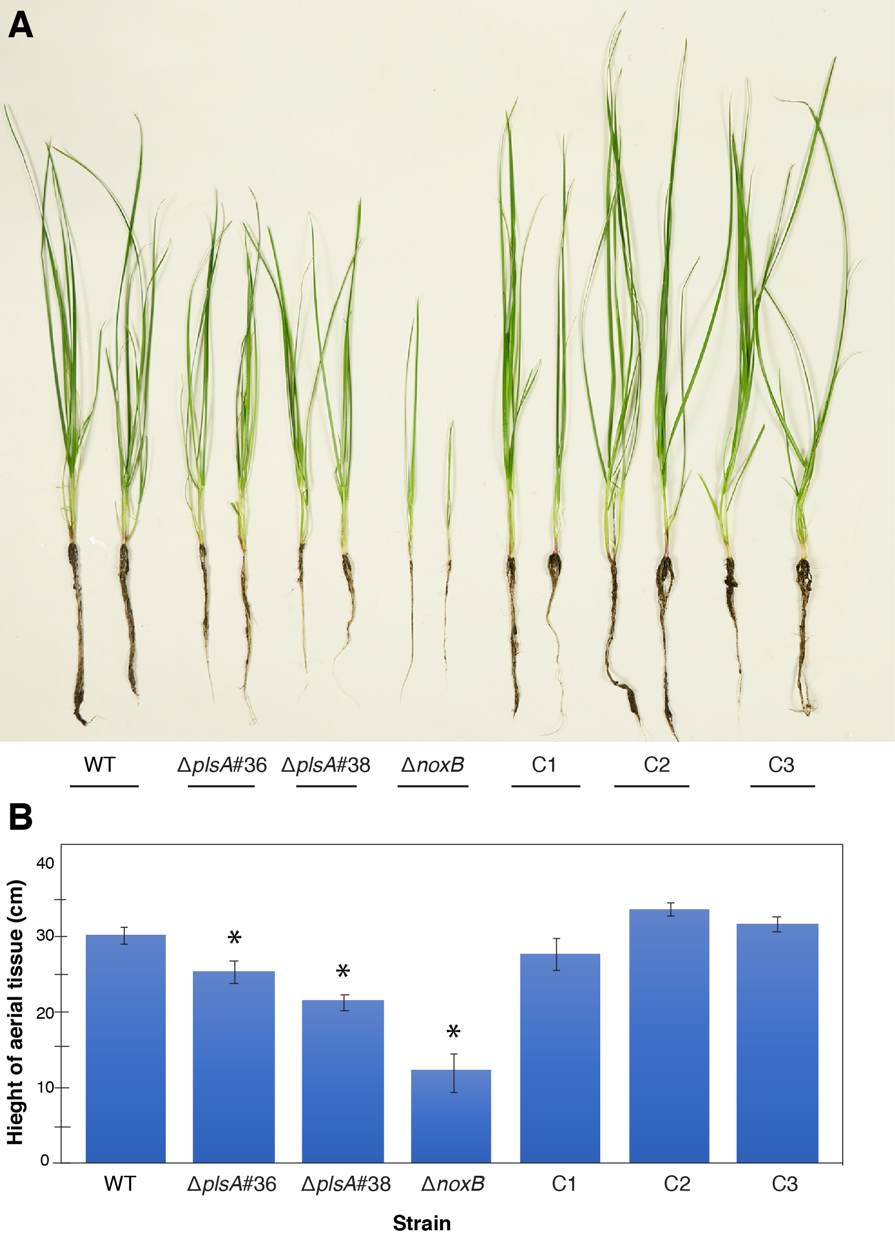
**

**Fig. S4** Complementation of ∆*plsA* defects *in planta.* (A) Host morphology in wild-type, ∆*plsA,* ∆*noxB* and ∆*plsA*/*plsA* (C1-3) associations at seven weeks post planting. (B) Height of the tallest infected tiller (wild-type, ∆*plsA* and ∆*plsA/plsA* associations n=14-18; ∆*noxB* associations n=3). An asterisk indicates significant differences from wild-type (*p* <0.05), as determined by One-Way ANOVA test.
