# Supplementary material for: A homologue of the fungal tetraspanin Pls1 is required for Epichloë festucae expressorium formation and establishment of a mutualistic interaction with Lolium perenne
Source: Mol Plant Pathol. 2019 Apr 22;20(7):961–75. doi: 10.1111/mpp.12805 (PMC6589725; doi:10.1111/mpp.12805)
Supplement: Supplementary file 6 — Table S2 Biological material. [file MPP-20-961-s006.docx]

| **Supplementary Table 2: Biological Material** |  |  |
| --- | --- | --- |
| **Biological material** | **Relevant characteristics** | **Reference** |
| **Yeast strains** |  |  |
| *S. cerevisiae* |  |  |
| PN2806 (FY834) | *MATa his3Δ200 ura3-52 leu2Δ1 lys2Δ202 trp1Δ63* | (Winston *et al.*, 1995) |
|  |  |  |
| **Fungal strains** |  |  |
| *Epichloë festucae* |  |  |
| PN2278 (Fl1) | Wild-type, isolated from host *Festuca longifolia* | (Young *et al.*, 2005) |
| PN2469 (∆*noxB*) | Fl1/Δ*noxB*::*PtrpC-nptII-TtrpC*; Gen^R^ | (Tanaka *et al.*, 2006) |
| PN3062 (∆*noxB*#1) | Fl1/Δ*noxB*::*PtrpC-nptII-TtrpC*; Gen^R^ | (Becker *et al.*, 2016) |
| PN3063(∆*noxB*#14) | Fl1/Δ*noxB*::*PtrpC-nptII-TtrpC*; Gen^R^ | (Becker *et al*., 2016) |
| PN3064 (∆*noxB*#15) | Fl1/Δ*noxB*::*PtrpC-nptII-TtrpC*; Gen^R^ | (Becker *et al*., 2016) |
| PN3247 (∆plsA#36) | Fl1/Δ*plsA:*:P*trpC*-*hph*; Hyg^R^ | This study |
| PN3248 (∆plsA#38) | Fl1/Δ*plsA*::P*trpC*-*hph*; Hyg^R^ | This study |
| PN3245 (Fl1::Sep3-GFP) | Fl1/pKG36; Hyg^R^ | This study |
| PN3249 (∆plsA#36 Sep3-eGFP) | Δ*plsA*#36/pKG36 pII99; Gen^R^, Hyg^R^ | This study |
| PN3250 (∆plsA#38 Sep3-eGFP) | Δ*plsA*#38/pKG36 pII99; Gen^R^, Hyg^R^ | This study |
| PN3256 (∆*plsA*#38/*plsA* C1) | Δ*plsA#*38/pKG34; Hyg^R^, Gen^R^ | This study |
| PN3257 (∆*plsA*#38/*plsA* C2) | Δ*plsA#*38/pKG34; Hyg^R^, Gen^R^ | This study |
| PN3258 (∆*plsA*#36/*plsA* C3) | Δ*plsA#*36/pKG34; Hyg^R^, Gen^R^ | This study |
|  |  |  |
| **Bacterial strains** |  |  |
| *E. coli* |  |  |
| PN1687 (pII99) | P*trpC*-*nptII*-T*trpC*; Amp^R^/Gen^R^ | (Namiki *et al.*, 2001) |
| PN1862 (pSF15.15) | P*trpC*-hph-T*trpC*; Amp^R^/Hyg^R^ | (Takemoto *et al.*, 2006) |
| PN413 (pRS426) | *ori*(f1)-*lacZ*-T7 promoter-MCS (*Kpn*I-*Sac*I)-T3 promoter-*lacI*-*ori*(pMB1)-Amp^R^-ori (2 micron), URA3, Amp^R^ | (Christianson T. W. *et al.*, 1992) |
| PN1994 (pPN94) | pSF14.10 containing P*tefA*-T*trpC* | (Takemoto *et al.*, 2006) |
| PN1199 (pAN7-1) | Hyg^R^ | (Punt *et al.*, 1987) |
| PN4149 (pCE60) | pRS426 containing 5'*plsA*-P*trpC*-*hph*-3'*plsA*; Amp^R^/Hyg^R^ | This study |
| PN4318 (pKG36) | pPN94 containing P*tefA*-*Sep3*-*eGFP*-T*trpC*; Amp^R^, Hyg^R^ | This study |
| PN4322 (pKG34) | pAN7-1 containing P*plsA-plsA*-T*plsA;* Amp^R^ | This study |
|  |  |  |
| **Plasmids** |  |  |
| pII99 | P*trpC*-*nptII*-T*trpC*; Amp^R^/Gen^R^ | (Namiki *et al*., 2001) |
| pSF15.15 | P*trpC*-*hph*-T*trpC*; Amp^R^/Hyg^R^ | (Takemoto *et al.*, 2006) |
| pRS426 | *ori*(f1)-*lacZ*-T7 promoter-MCS (*KpnI*-*SacI*)-T3 promoter-*lacI*-*ori*(pMB1)-Amp^R^-*ori* (2 micron), URA3, Amp^R^ | (Christianson T. W. *et al*., 1992) |
| pPN94 | pSF14.10 containing P*tefA*-T*trpC* | (Takemoto *et al*., 2006) |
| pAN7-1 | Hyg^R^ | (Punt *et al*., 1987) |
| pCE60 | pRS426 containing 5'*plsA*-P*trpC*-*hph*-3'*plsA*; Amp^R^/Hyg^R^ | This study |
| pKG36 | pPN94 containing P*tefA*-*Sep3*-*eGFP*-T*trpC*; Amp^R^, Hyg^R^ | This study |
| pKG34 | pAN7-1 containing P*plsA-plsA*-T*plsA;* Amp^R^ | This study |

**REFFERENCES**

**Becker, M., Becker, Y., Green, K. and Scott, B.** (2016) The endophytic symbiont *Epichloë festucae* establishes an epiphyllous net on the surface of *Lolium perenne* leaves by development of an expressorium, an appressorium-like leaf exit structure. *New Phytol,* **211,** 240-254.

**Christianson T. W., Sikorski R. S., Dante M., Shero J. H. and Hieter P.** (1992) Multifunctional yeast high-copy-number shuttle vectors. *Gene,* **110,** 119-122.

**Namiki, F., Matsunaga, M., Okuda, M., Inoue, I., Nishi, K., Fujita, Y.*, et al.*** (2001) Mutation of an arginine biosynthesis gene causes reduced pathogenicity in *Fusarium oxysporum* f. sp. *melonis*. *Mol Plant Microbe Interact,* **14,** 580-584.

**Punt, P. J., Oliver, R. P., Dingemanse, M. A., Pouwels, P. H. and Hondel, C. A. M. J. J. v. d.** (1987) Transformation of *Aspergillus* based on the hygromycin B resistance marker from *Escherichia coli*. *Gene,* **56,** 117-124.

**Takemoto, D., Tanaka, A. and Scott, B.** (2006) A p67Phox-like regulator is recruited to control hyphal branching in a fungal–grass mutualistic symbiosis. *Plant Cell,* **18,** 2807-2821.

**Tanaka, A., Christensen, M. J., Takemoto, D., Park, P. and Scott, B.** (2006) Reactive oxygen species play a role in regulating a fungus–perennial ryegrass mutualistic interaction. *Plant Cell,* **18,** 1052-1066.

**Winston, F., Dollard, C. and Ricupero-Hovasse, S. L.** (1995) Construction of a set of convenient *Saccharomyces cerevisiae* strains that are isogenic to S288C. *Yeast,* **11,** 53-55.

**Young, C. A., Bryant, M. K., Christensen, M. J., Tapper, B. A., Bryan, G. T. and Scott, B.** (2005) Molecular cloning and genetic analysis of a symbiosis-expressed gene cluster for lolitrem biosynthesis from a mutualistic endophyte of perennial ryegrass. *Mol Genet Genomics,* **274,** 13-29.
