# Supplementary material for: A homologue of the fungal tetraspanin Pls1 is required for Epichloë festucae expressorium formation and establishment of a mutualistic interaction with Lolium perenne
Source: Mol Plant Pathol. 2019 Apr 22;20(7):961–75. doi: 10.1111/mpp.12805 (PMC6589725; doi:10.1111/mpp.12805)
Supplement: Supplementary file 7 — Table S3 Primers used in this study. [file MPP-20-961-s007.docx]

| **Supplementary Table 3:** Primers used in this study. | | |  |
| --- | --- | --- | --- |
|  |  | |  |
| **Primer** | **Sequence** | **Used for** | |
| hph-F | AGCTTGGAACTGATATTGAAGG | Hygromycin fragment for pCE60 | |
| hph-R | CTATTCCTTTGCCCTCGGACG | Hygromycin fragment for pCE60 | |
| pRS426-plsA-F | GTAACGCCAGGGTTTTCCCAGTCACGACGGATCCTGACAAGACCTTTCTACCAGC | *plsA* 5' fragment for pCE60 | |
| plsA-hph-R | CCAGCACTCGTCCGAGGGCAAAGGAATAGTTGGAGAGTGTGAAAGACCAG | *plsA* 5' fragment for pCE60 | |
| hph-plsA-F | AAATGCTCCTTCAATATCAGTTCCAAGCTTCAGCCATATCAAGCTTCTCC | *plsA 3*' fragment for pCE60 | |
| plsA-pRS426-R | GCGGATAACAATTTCACACAGGAAACAGCGAATTCCCACGGCATCAACCTGTAACG | *plsA 3*' fragment for pCE60 | |
| pls5 | CGAACTCTGGTCTTTCACAC | *plsA* knock out screening primer | |
| pls6 | TATGGACAAGTTGGAGAAGC | *plsA* knock out screening primer | |
| KG150 | AGGGTTTTCCCAGTCACGACATAGCCAGCTAGGATTGC | *plsA* fragment for pKG34 | |
| KG151 | CAATTTCACACAGGAAACAGCGTTCCCTGAAGATGACGG | *plsA* fragment for pKG34 | |
| pRS426_F | GCTGTTTCCTGTGTGAAATTG | pAN7-1 fragment for pKG34 | |
| pRS426_R | GGGTTTTCCCAGTCACGAC | pAN7-1 fragment for pKG34 | |
| ptefR | CTAGAGGTTTGACGGTGATG | eGFP for pKG36 | |
| gfpF | GGTGCTGGTGCTGGTGCT | eGFP for pKG36 | |
| KG158 | CATCACCGTCAAACCTCTAGTGCTACTTCCTCATCGAC | Sep3 for pKG36 | |
| KG155 | AGCACCAGCACCAGCACCACGCAGTGAGAAACCCTTTCGC | Sep3 for pKG36 | |
