# Supplementary material for: A homologue of the fungal tetraspanin Pls1 is required for Epichloë festucae expressorium formation and establishment of a mutualistic interaction with Lolium perenne
Source: Mol Plant Pathol. 2019 Apr 22;20(7):961–75. doi: 10.1111/mpp.12805 (PMC6589725; doi:10.1111/mpp.12805)
Supplement: Supplementary file 7 [file MPP-20-961-s005.docx]

Supplementary Table 1: Survival and infection rates of wild-type, mutant and complemented strains

**Experiment 1**	Planted	Survived (S)	Infected (I)	% S	% I
Fl1	56	27	16	48%	59%
Δ*plsA*#36	56	21	7	38%	33%
Δ*plsA*#38	56	23	9	41%	39%
Δ*noxB*	56	12	3	21%	25%

**Experiment 2**	Planted	Survived (S)	Infected (I)	% S	% I
Fl1	56	24	20	43%	83%
Δ*plsA*#36	56	24	14	43%	58%
Δ*plsA*#38	63	23	17	37%	74%
Δ*noxB*	48	5	3	10%	60%

**Experiment 3**	Planted	Survived (S)	Infected (I)	% S	% I
Fl1	56	37	24	66%	65%
Δ*plsA*#36	56	18	12	32%	67%
Δ*plsA*#38	56	23	13	41%	57%
Δ*noxB*	52	14	3	27%	21%
C1	46	22	9	48%	41%
C2	40	32	19	80%	59%
C3	52	39	27	75%	69%
